# Synthesis of Refractory High-Entropy Alloy WTaMoNbV by Powder Bed Fusion Process Using Mixed Elemental Alloying Powder

**DOI:** 10.3390/ma15124043

**Published:** 2022-06-07

**Authors:** Tomer Ron, Avi Leon, Vladimir Popov, Evgeny Strokin, Dan Eliezer, Amnon Shirizly, Eli Aghion

**Affiliations:** 1 Department of Materials Engineering, Ben-Gurion University of the Negev, Beer-Sheva 8410501, Israel; avileon12@gmail.com (A.L.); deliezer@bgu.ac.il (D.E.); a.shirizly@gmail.com (A.S.); egyon@bgu.ac.il (E.A.); 2 Institute of Metals, Technion, Haifa 3200003, Israel; vvp0604@gmail.com (V.P.); strokin@trdf.technion.ac.il (E.S.)

**Keywords:** additive manufacturing, laser powder bed fusion, high-entropy alloys, refractory alloys

## Abstract

The growing interest in refractory high-entropy alloys (HEAs) in the last decade is mainly due to their thermal stability, outstanding mechanical properties, and excellent corrosion resistance. However, currently HEAs are still not considered for use as common structural materials due to their inherent drawbacks in terms of processing and machining operations. The recent progress witnessed in additive manufacturing (AM) technologies has raised the option of producing complex components made of HEAs with minimal machining processes. So far, this could be achieved by using pre-alloyed powders of HEAs that were mainly produced by a conventional arc melting furnace (AMF) in the form of small compounds that were transformed into powder via a gas atomization process. To significantly reduce the production cost, the present study aims to analyze the ability to synthesize HEA WTaMoNbV via a laser powder bed fusion (LPBF) process using mixed elemental alloying powder as the raw material. For comparison, a counterpart alloy with the same chemical composition was analyzed and produced by an AMF process. The microstructures of the tested alloys were examined by scanning electron microscopy (SEM), transmission electron microscopy (TEM) and X-ray diffraction (XRD) analyses. The physical properties were evaluated in terms of density and mechanical strength, while the electrochemical behavior was assessed by potentiodynamic polarization analysis. The results disclosed similarities in microstructure, physical properties and electrochemical behavior between HEA WTaMoNbV manufactured by the proposed LPBF process and its counterpart alloy produced by an AMF process.

## 1. Introduction

The attractiveness of refractory high-entropy alloys (HEAs) as structural materials stems from their outstanding mechanical properties, thermal stability, wear resistance and corrosion performance in hostile environments [1,2,3,4,5]. Recently, refractory high-entropy alloys have received significant attention in relation to high-temperature application in aerospace, defense, and nuclear power generation industries [6]. In fact, they were designated to replace traditional Ni-based super-alloys in applications such as gas turbines, heat exchangers, aerospace propulsion components, rocket engine nozzle [7]. HEAs are multicomponent systems that are composed from a small number of principal elements. They are usually composed from at least five elements with individual atomic concentrations that range between 5% and 35%. HEAs can produce solid phase solutions, mainly due to their configurational entropy and ability to be adequately synthesized [8,9,10]. Conventionally, HEAs are mainly produced in an arc melting furnace (AMF), but they may also be produced by mechanical alloying, plasma spark sintering and physical vapor deposition [11]. Refractory HEAs are particularly difficult to process due to their high melting temperature, high hardness, natural tendency to oxidize and toughness in machining operations [12]. Hence, there is great interest in finding ways to produce these alloys by additive manufacturing (AM) technologies, which could enable the production of complex components with minimal machining processes [13,14,15].

The concept of using AM technologies to produce HEA components was initially introduced by Kunce et al. in 2013 [16], and was later investigated by other researchers [17,18,19,20]. In general, the research relating to AM of HEA from pre-alloyed powder obtained mainly by gas atomization [21,22,23] can be divided into three main categories: (i) powder bed fusion, including laser powder bed fusion (LPBF) [24,25,26] and electron beam melting (EBM) [27,28]; (ii) direct energy deposition using blown powder deposition (BPD) [29,30,31]; and (iii) binder jetting [32]. The use of spherical pre-alloyed powders tends to improve the row material flowability as required in powder bed fusion processes [33].

In parallel, some initial AM efforts were dedicated to attempting to produce HEA from a mix of elemental alloying powder composed of the following material systems: Al0.5CrMoNbTa0.5 [34,35], NbMoTaW [8] and AlCoFeNiSmTiVZr [1]. However, the physical properties obtained in those studies were significantly inferior compared to AM samples produced from pre-alloyed powders.

Senkov et al. [36] reported that they succeeded in producing the refractory HEA WTaMoNbV considered in this research by using the conventional vacuum AMF. Their results demonstrated excellent mechanical properties and thermal stability at high temperatures (1200 °C). A similar HEA composition in the form of WTaMoNb (without vanadium) was also produced by an AM process from pre-alloyed material. Dobbelstein et al. [12] were the first to produce this alloy with direct deposition technology in the form of a few consecutive layers in 2016. Since then, only three further studies have been published [1,7,11], all with the objective of achieving adequate solid solution synthesis with a BCC crystal structure.

The main challenge related to the composition of the HEA WTaMoNbV that was selected mainly due to the high melting temperature of its elemental ingredients relates to proper selection of the AM process parameters that can adequately synthesize this alloy. This challenge is mainly due to the significant diversity in the thermal and physical properties of the alloying elements, as shown in Table 1. For example, the differences between the melting temperatures of tungsten, on the one hand, and niobium and vanadium, on the other, are 945 °C and 1512 °C, respectively, while the differences between their densities are 10.68 and 13.14 gr/cm^3^, respectively. The aim of this study is to evaluate the possibility of synthesizing refractory HEA WTaMoNbV via a LPBF process using mixed elemental alloying powder as the raw material. This evaluation includes the preparation of a counterpart alloy with the same chemical composition via a conventional AMF process to serve as a reference. The ability to produce refractory HEA by LPBF will make it possible to create complex geometry at reasonable cost.

## 2. Materials and Methods

### 2.1. Preparation of HEA WTaMoNbV Samples from Mixed Elemental Powder

Test specimens and a technology demonstrator component in the form of a turbine blade were produced from HEA WTaMoNbV by an LPBF process [37] using a mixed powder of the alloying elements. The LPBF facility included an EOS EOSINT M290 system equipped with a 400 W Nd-YAG laser and pure argon as the protective gas atmosphere. The powder used to produce the HEA WTaMoNbV was composed from mixed powders of the alloying elements (W, Ta, Mo, Nb and V) with equal atomic amounts (20% each). The detailed chemical composition of the high-purity elemental powders in terms of ppm impurities is shown in Table 2. The mixed powder was obtained by intensive stirring of the pure elemental powders within a rotating sealed chamber for 6 h using a turning rate of 23 rpm until a homogenous mixed powder was obtained.

In order to attain adequate properties from the LPBF process, a large number of AM sessions were carried out and designated as primary trials, intermediate trials and advanced trials. The printing parameters of these trials were varied as follows: laser power 160–244 W, spot size 80 µm, scanning speed 300–700 mm/s, hatch spacing 0.10–0.12 mm, building layer thickness 0.3 mm, energy density 38.1–162.7 [J/mm^3^] and final obtained densities 38–95 [%], as shown in Table 3. The scanning direction was rotated 67 degrees after each successive layer to attain optimal densification. The energy density was calculated according to the following equation:

$$E=\frac{P}{V\cdot h\cdot t}$$

where *E*—energy density, *P*—laser power, *V*—scanning speed, *h*—hatch spacing and *t*—building layer thickness.

As a reference, a counterpart HEA was produced with conventional technology using an AMF with the same mixed elemental powder that was used for the LPBF process. The melting current and melting range capabilities of the AMF (Edmund Buhler GmbH MAM1E-H180T) were 5–180 A and up to 4000 °C, respectively, and high-purity argon was used as the protective gas atmosphere. The amount of the mixed elemental powder loaded to the AMF was 12 g. Prior to this loading the mixed powder was compacted at room temperature up to a green density of about 50% to obtain an adequately consolidated substance.

### 2.2. Microstructure Analysis

Microstructure analysis was carried out by scanning electron microscopy (SEM) (SEM-JEOL 5600, JEOL Ltd., Tokyo, Japan), equipped with an energy-dispersive X-ray spectroscopy (EDS) sensor [38] for localized chemical composition detection. The metallographic preparation included polishing up to 0.04 µm. The presence of secondary phases was evaluated by X-ray diffraction (XRD) analysis using a RIGAKU-2100H X-ray diffractometer with CuKα [39]. The diffraction parameters were 40 KV/30 mA and a scanning rate of 2°/min. For high-resolution observation, transmission electron microscopy (TEM) characterization was carried out using an analytical electron microscope (JEOL JEM-2100F, Jeol Ltd., Tokyo, Japan) facility operating at 200 kV. This observation included bright field (BF) analysis, selected area electron diffraction (SAED), and EDS. For microstructural investigation at the center of indentations, electron-transparent cross-section lamella specimens were meticulously prepared with a dual-beam focused ion beam microscope (FEI, Verios-460 L, Hillsboro, OR, USA).

### 2.3. Assessment of Physical Properties and Environmental Behavior

The physical properties of the additively manufactured HEA WTaMoNbV samples were evaluated in terms of mechanical properties and density. For statistical reference, the mechanical properties were measured 5 times for each test, including Vickers hardness, measured by SM1016 TecQuipment Ltd., Nottingham, UK, and compression strength using a Hounsfield H25KT testing machine with a crosshead-speed of 0.5 mm/min. The environmental behavior in terms of electrochemical analysis by potentiodynamic polarization was carried out using a Bio-Logic SP-200 potentiostat (BioLogic Science Instruments, Seyssinet-Pariset, France), equipped with Ec-Lab software—V11.18 [40]. This assessment was implemented using a standard three-electrode cell with saturated calomel (SCE) as the reference electrode. The scanning rate of the potentiodynamic polarization analysis was 0.5 mV/s and the corrosive environment was in the form of a 3.5% NaCl solution at ambient temperature. Prior to the electrochemical analysis, the test samples were cleaned in an ultrasonic bath for 5 min, followed by washing with alcohol and air drying.

## 3. Results

### 3.1. Powder Analysis

**Figure 1:**
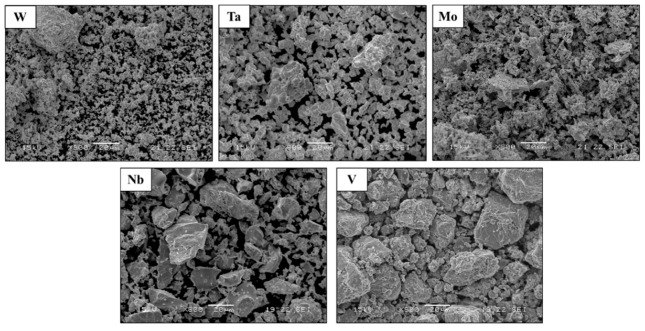
General views of the elemental powders used to produce the HEA WTaMoNbV by LPBF process.

General views of the different elemental powders used to produce the mixed HEA WTaMoNbV powder as the raw material for the LPBF process are shown in Figure 1. Although the size distribution of the different particles was between 20–70 µm, the average particle size was between 40–50 µm, as shown in Table 4. It should be pointed out that the irregularity in the shapes of the different alloying particles assists in attaining a more unified and compacted powder mix.

### 3.2. Chemical Composition Analysis and Macroscopic Structure Examination

The average chemical compositions of HEA WTaMoNbV produced by the LPBF process and its counterpart alloy produced by conventional AMF are shown in Table 5. As expected, both alloys showed similar chemical composition with relatively small deviations (less than 3.9%at) from the designated composition of 20% for each alloying element. The chemical composition was measured in five different areas in the sample. The highest standard deviation of Ta (21%) shows non-homogeneous compositions of the different elements.

**Figure 2:**
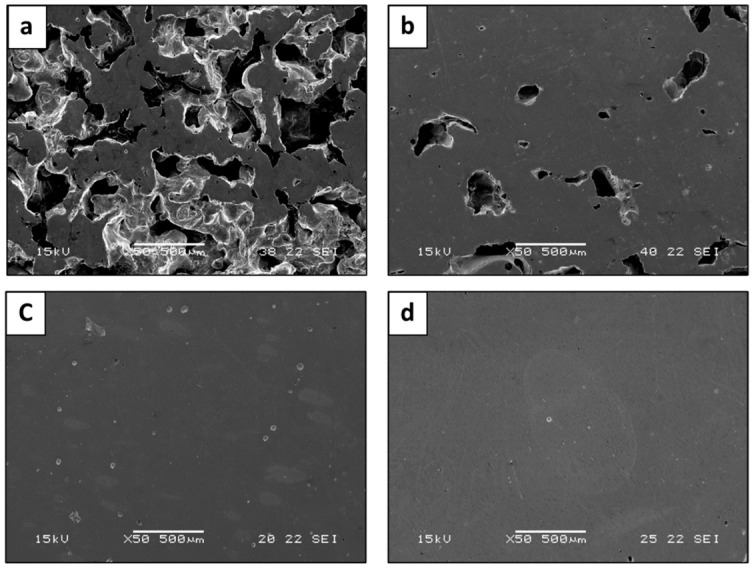
Macroscopic structure of HEA WTaMoNbV obtained by various LPBF trials and AMF processes: (**a**) LPBF—Primary trials, (**b**) LPBF—Intermediate trials, (**c**) LPBF—Advanced trials, (**d**) counterpart alloy obtained by AMF process.

The typical macrostructure of HEA WTaMoNbV produced by various LPBF trials is shown in Figure 2a–c along with the macrostructure of the counterpart alloy produced by AMF, shown in Figure 2d. This reveals a significant difference between the primary LPBF trials and the advanced LPBF trials in terms of inherent defects, mainly in the form of lack-of-fusion and porosity [41]. Apparently, the relatively large amount of binding defects found in the primary LPBF trials can be related to inadequate fusion of consecutive powder layers [42]. This can be attributed to the insufficient local energy density that is required for proper layer fusion [43]. The differences between the primary, on the one hand, and the intermediate and advanced LPBF trials, on the other, in terms of densities, were 38% and 95%, respectively. Comparatively, the macrostructure of the samples obtained by the advanced LPBF trials was relatively similar to that of the counterpart alloy obtained by the AMF process. Hence, all further analyses introduced by this study only relate to samples produced by the advanced LPBF trials.

### 3.3. Microstructure Analysis

**Figure 3:**
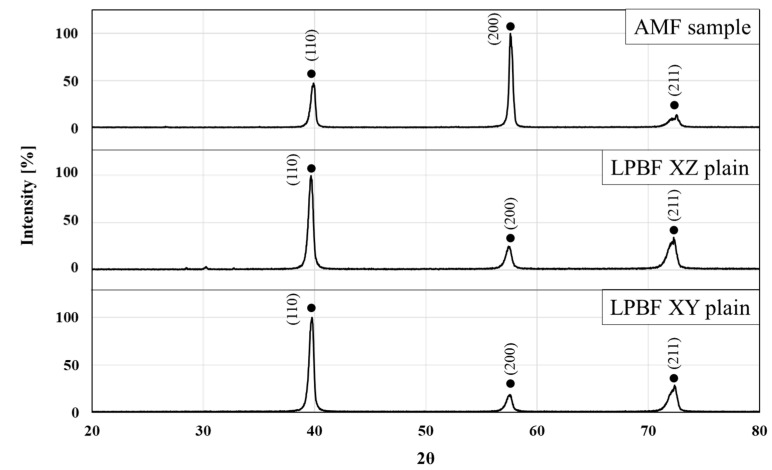
X-ray diffraction analysis of HEA WTaMoNbV produced by AMF and LPBF processes.

XRD analysis results for HEA WTaMoNbV produced by the LPBF process and its counterpart AMF alloy are shown in Figure 3. This reveals that the diffraction patterns of both alloys were similar, apart from differences in peak intensities that could be related to the relatively preferred orientation of the LPBF samples. Both alloys were composed of a single solid phase solution with a BCC crystal structure, as obtained by Zhang et al. [44], and had a similar lattice parameter of 3.2 Å. In addition, small peak shifts were observed in the LPBF samples in comparison to the AMF sample. The shifting of these peaks can be related to normal residual stresses that are produced during the additive manufacturing process.

**Figure 4:**
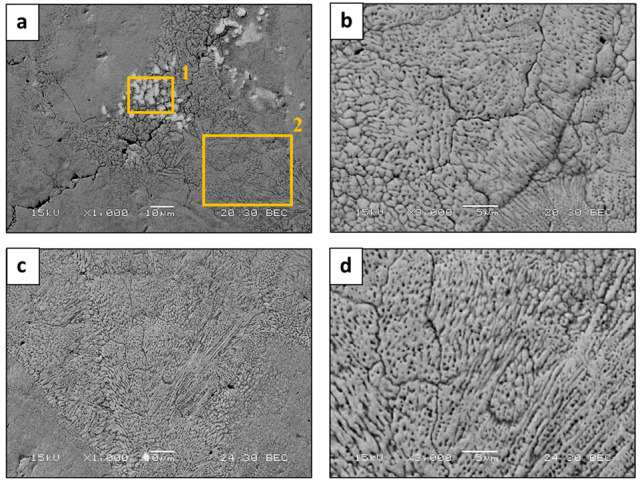
Microstructure of HEA WTaMoNbV produced by LPBF in terms of SEM backscattered electron mode: (**a**,**b**) XY plane, (**c**,**d**) XZ plane.

Typical microstructures of HEA WTaMoNbV produced by the LPBF process are shown in Figure 4. In general, this reveals the presence of a single solid phase solution with a relatively fine grain size (average size 10 µm). In addition, it shows the differences between the microstructure in the XY plane (printing path—top cross section view) and in the XZ plane (printing direction—front cross section view). The difference in the diffraction intensities between the XY plane and the XZ plane is indicative of preferred orientation due to epitaxial solidification, as expected from additively manufactured processes. However, the XRD results showed no significant sign of preferred orientation between the printing path and the printing direction. Furthermore, the zoom-out view (Figure 4a) along with local chemical composition analysis (Table 6) exposed differences in the distribution of tungsten. This was manifested by a relatively high amount of W (37.6%) in bright grains shown in Area 1 compared to only 15% of W in a neighboring site (Area 2). This can be attributed mainly to the relatively high melting temperature and high density of W compared to other alloying elements. In addition, minor cracks were observed in some parts of the LPBF sample, with a maximum crack density of $6.4\times 10^{-7}{\;\mu m}^{-2}$.

**Figure 5:**
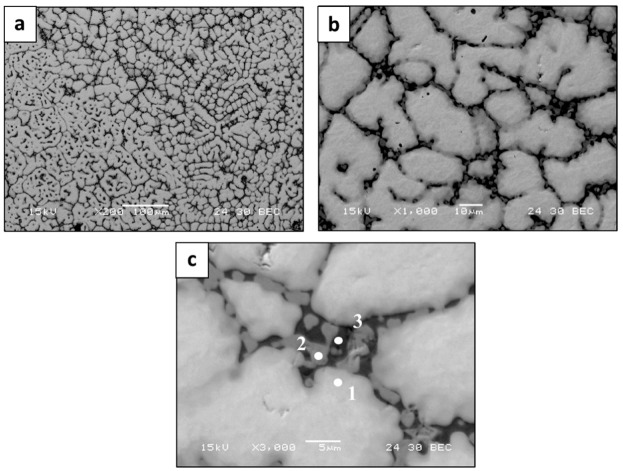
Microstructure by SEM backscattered electron mode of HEA WTaMoNbV produced by AMF process: (**a**) general appearance, (**b**) close-up view, and (**c**) magnified view of grain boundaries.

**Figure 6:**
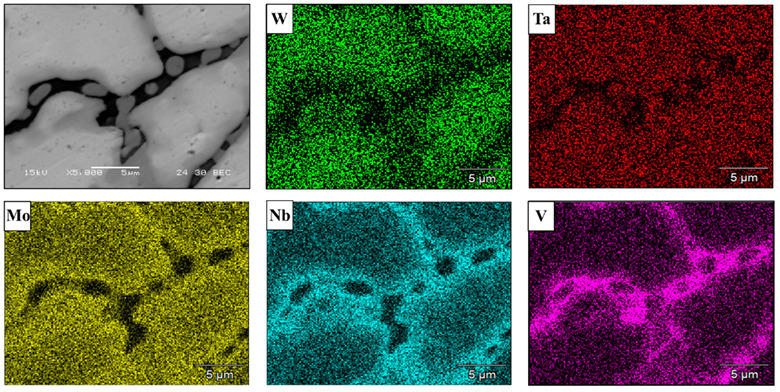
High-magnification image and corresponding EDS maps of elemental ingredients composing HEA WTaMoNbV alloy produced by AMF process.

Typical microstructures of the counterpart alloy produced by the AMF process are shown in Figure 5. Although the counterpart alloy also presents a single solid phase solution structure, its grain size was significantly increased compared to the LPBF samples (average size 25 µm). In addition, Figure 5c, along with the local composition analysis (Table 7), reveals a significant depletion of W at grain boundaries, accompanied by a substantial increase in V in this region. This phenomenon can be attributed to the relatively low melting temperature of V, which consequently solidified at the end of the solidification process, and hence was found to prefer grain boundaries. As supporting evidence, the phase compositions at high magnification with corresponding EDS maps are shown in Figure 6. This clearly demonstrates that the grain boundary mostly contains V and Nb, with relatively low concentration of W.

**Figure 7:**
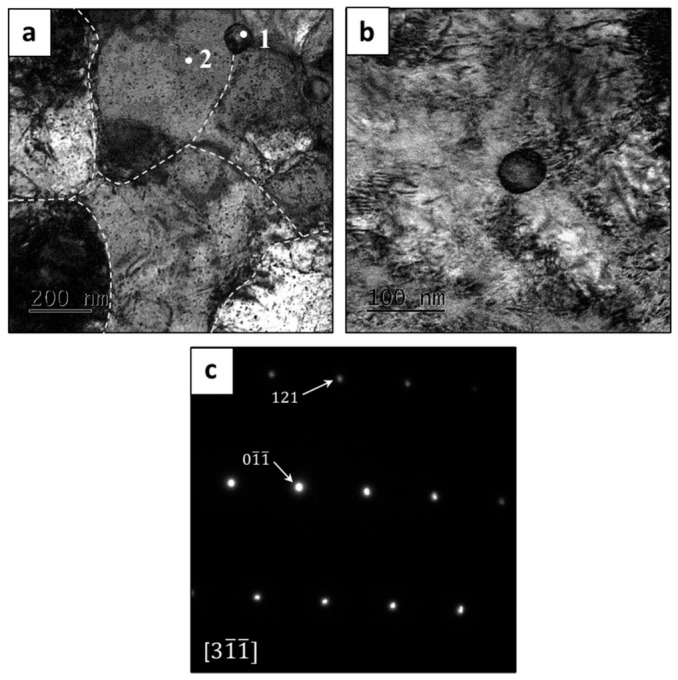
TEM analysis of HEA WTaMoNbV produced by LPBF process: (**a**,**b**) bright field (BF), (**c**) electron diffraction image of the alloy matrix.

The general microstructure of HEA WTaMoNbV produced by the LPBF process, as obtained by TEM analysis, is shown in Figure 7a. This high-resolution observation demonstrates the presence of sub-grains, with a size of about 500 nm, that compose the alloy matrix. Furthermore, evidence of high-density dislocations was also observed, and is probably due to common internal stresses inherently generated by the LPBF process. In addition, relatively small precipitates with a size of 60–80 nm and with a nearly spherical shape were dispersed along the intergranular boundaries, as shown in Figure 7b. It should be pointed out that this spherical precipitate was not identified by the XRD analysis, probably due to its relatively reduced amount (less than 2%). The chemical composition of those precipitates included a large amount of V (33%at) and a relatively depleted amount of W (7.9%at), as shown in Table 8. This observation complies with the outcome of the SEM analysis, from which it was concluded that the relatively low melting temperature of V makes this element more favorable to solidification at grain boundaries during the end stage of the solidification process. The electron diffraction image shown in Figure 7c corroborates the presence of a BCC crystal structure with Im3m symmetry. However, the precipitate structure was not decoded, due to the difficulty in finding different ordinations of the particle and the lack of literature on this aspect.

### 3.4. Physical Properties Assessment

The examined physical properties of HEa WTaMoNbV produced by the LPBF process were similar to those of the counterpart AMF alloy, being about 1.6% lower in terms of density, while its hardness was slightly reduced, as shown in Table 9.

**Figure 8:**
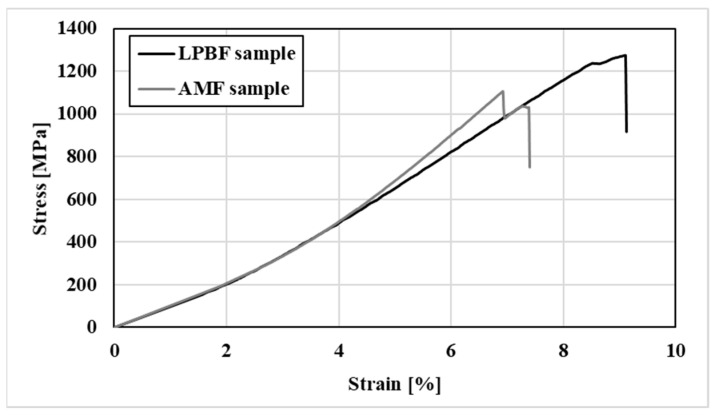
Compressive stress–strain curves of HEA WTaMoNbV produced by LPBF and AMF processes.

The mechanical properties of HEa WTaMoNbV produced by LPBF and AMF as expressed in terms of its compressive stress–strain curve are shown in Figure 8. The ultimate compressive strength of the LPBF sample was slightly higher than the counterpart AMF sample, as shown in Table 10. Both alloys presented relatively brittle compressive stress–strain curves with significantly reduced elongation. For reference consideration, a similar result for the AMF sample was presented by Senkov et al. [36].

**Figure 9:**
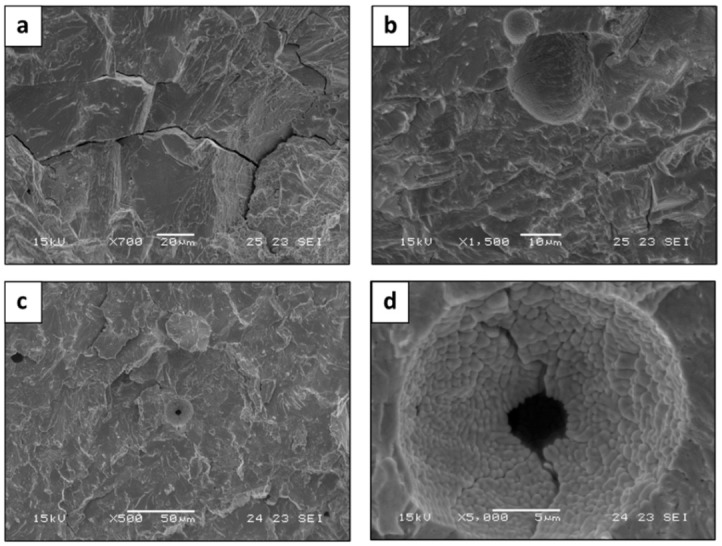
Fractography analysis of HEA WTaMoNbV produced by LPBF process: (**a**,**b**) trans-granular fracture, (**c**,**d**) fracture surface with typical LPBF defects.

**Figure 10:**
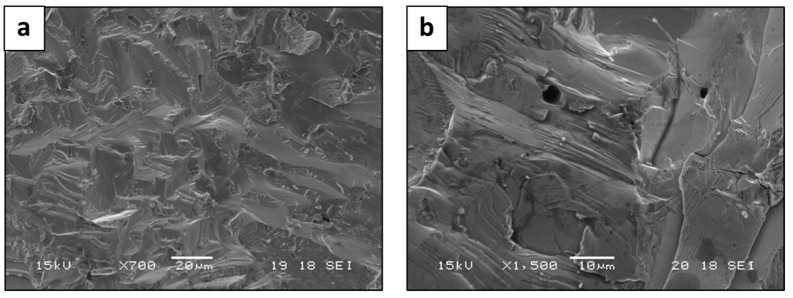
Fractography analysis of HEA WTaMoNbV produced by AMF process, showing trans-granular fracture.

The fracture failure analysis of the LPBF and AMF samples obtained by SEM revealed a relatively brittle fracture with a transgranular morphology, as shown in Figure 9a,b and Figure 10, respectively. In addition, Figure 9c,d indicates that the fracture surface contains typical LPBF defects, such as porosity and un-melted powder particles that induce brittleness. The higher compressive strength and more brittle fracture of the LPBF alloy may be related to differences in the microstructure and solidification defects and in the finer grain size.

### 3.5. Environmental Behavior Assessment

**Figure 11:**
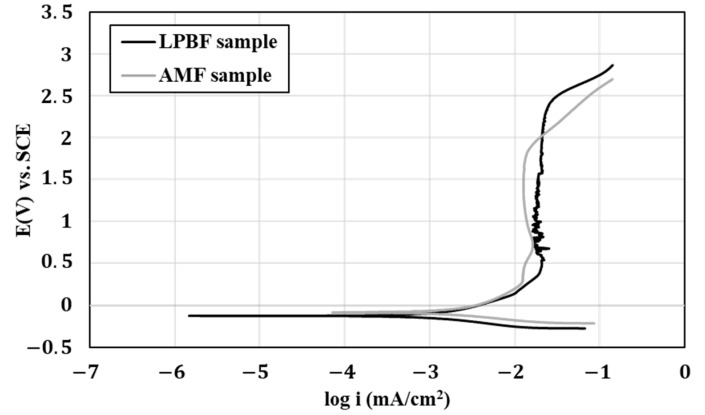
Potentiodynamic polarization analysis of HEA WTaMoNbV produced by LPBF versus its counterpart AMF alloy.

**Figure 12:**
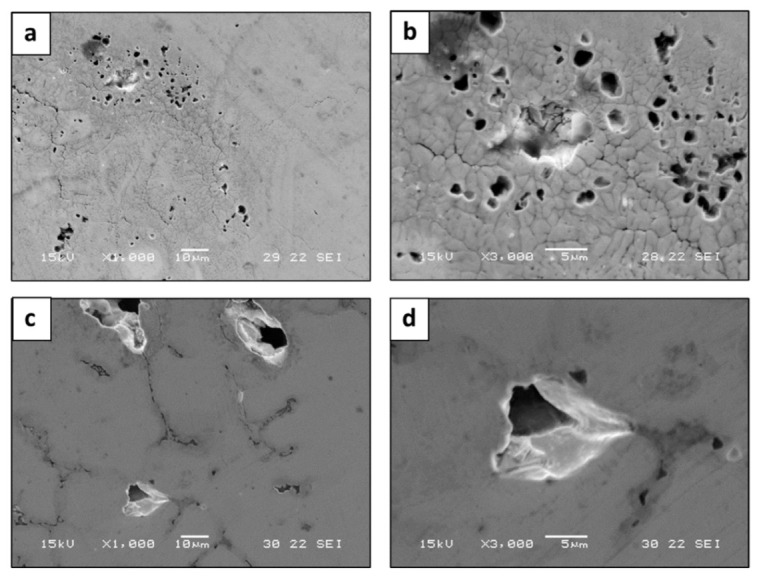
General appearance of localized corrosion attack: (**a**,**b**) HEA WTaMoNbV produced by LPBF process, (**c**,**d**) counterpart AMF alloy.

The environmental behavior of HEA WTaMoNbV obtained by the LPBF process versus that of its counterpart AMF alloy in terms of potentiodynamic polarization analysis is shown in Figure 11, along with the derived electrochemical parameters introduced in Table 11. This indicates that both alloys showed excellent corrosion resistance with a typical active–passive transition mode [45,46]. In addition, due to the relatively increased roughness of the LPBF alloy, its passivation stability manifested by current density fluctuations was reduced. Regarding the passivation current, although the counterpart AMF alloy showed relatively reduced current density, the break potential of the LPBF alloy that signified the transition to the transpassive state and breakdown of the passive film [47] was higher. This basically means that although the passivation of the AMF alloy is easier to obtain, its stability is reduced. As a result, the localized corrosion attack of the LPBF alloy in terms of pit dimensions was reduced compared to the AMF alloy, as shown in Figure 12.

## 4. Discussion

This basic feasibility analysis addresses the question of whether HEA WTaMoNbV can be adequately produced via the LPBF process using mixed elemental alloying powder as the raw material. In fact, the more fundamental question is whether the proposed LPBF process is capable of adequately synthesizing the alloying elements of HEA [12] in a similar manner to that obtained using a conventional AMF process [36].

The attained results clearly demonstrated that the macrostructure and phase composition of samples produced by LPBF were similar to those obtained by its counterpart AMF alloy. In both cases, the alloys were composed of a single solid-phase solution with a BCC crystal structure with similar lattice parameters, as expected according to Kang et al. [48]. In terms of microstructure, both alloys showed differences in the distribution of tungsten and an increased amount of vanadium at grain boundaries. This can mainly be related to the relatively low melting temperature of vanadium (1910 °C) versus tungsten (3422 °C), which consequently causes vanadium to solidify at grain boundaries during the final phase of the solidification process. Additional reasons for the elemental distribution in the case of the LPBF process may be related to differences in the shape and size of the powder particles. Their irregular size can affect the laser beam operation differently, and consequently influence the solidification process. This may relate, for example, to the relatively Ta size deviation (standard deviation of 20%). The combination of the high melting temperature (3017 °C) of Ta and the relatively large shape deviation can lead to uneven distribution. Hence it is believed that using spherical powders may improve the flowability of the powder and consequently increase homogeneity. The physical properties in terms of density as well as the mechanical properties in both the LPBF alloy and its counterpart AMF alloy were similar. The relatively increased compressive strength of the LPBF sample could be related to the relatively reduced grain size, due to inherent rapid solidification characteristics. In terms of environmental behavior, both alloys showed outstanding corrosion resistance and adequate passivation characteristics, as expected [1,5,7,11]. Nevertheless, the small differences in the corrosion mechanism of the two alloys related to the relatively non-homogeneous characteristics of the LPBF sample and inherent printing defects. This was visually manifested by the relatively increased number of small pits in the LPBF alloy compared to the counterpart AMF alloy, although the total mass loss of the two alloys were similar.

Altogether, this study was able to basically analyze and demonstrate the feasibility of producing HEA WTaMoNbV by an LPBF process from mixed elemental alloying powder. However, additional experimental work is required to further optimize the printing parameters to obtain improved properties and microstructure characteristics, mainly in terms of printing defects. This may also include post heat treatments that may upgrade the homogeneity of the LPBF alloy, as well as its synthesis characteristics.

## 5. Conclusions

- The synthesis of HEA WTaMoNbV via the laser powder bed fusion (LPBF) process using mixed elemental alloying powder was demonstrated as feasible because of its similar microstructure, physical properties and electrochemical behavior compared to its counterpart alloy produced by the conventional arc melting furnace (AMF) process.
- In both cases, the alloys were composed of a single solid phase solution, with a BCC crystal structure with similar lattice parameters.
- Further experimental work is needed to continue to optimize the printing parameters of the LPBF process along with possible a post-heat-treatment process to rectify inherent printing defects.

## Figures and Tables

**Table 1:** Typical physical properties of the considered refractory element.

	W	Ta	Mo	Nb	V
**Melting point (°C)**	3422	3017	2623	2477	1910
**Density (gr/cm^3^)**	19.25	16.65	10.28	8.57	6.11

**Table 2:** Chemical composition of the pure elemental powders in terms of impurities [ppm].

	C	N	O	Mg	Al	Si	P	S	Ca	Ti	V	Cr	Fe	Ni	Cu	Se	Nb	Mo	Sn	Sb	Ta	W
**Tungsten**	25	20	10	85	10	13	55	-	15	-	-	-	30	50	35	20	-	30	7	18	-	**Bal.**
**Tantalum**	-	-	-	-	-	17	-	-	-	10	-	-	14	18	-	-	-	-	-	-	**Bal.**	12
**Molybdenum**	35	16	50	2	3	9	10	10	-	-	-	-	38		3	-	-	**Bal.**	-	-	-	80
**Niobium**	-	-	-	<1	38	10	48	29	-	-	-	10	18	9	<1	-	**Bal.**	8	<1	-	-	72
**Vanadium**	-	-	1000	-	90	200	-	-	-	-	**Bal.**	70	1200	-	-	-	-	-	-	-	-	-

**Table 3:** Printing parameters of LPBF trials.

	Laser Power [W]	Scanning Speed [mm/s]	Hatch Spacing [mm]	Layer Thickness [mm]	Energy Density [J/mm^3^]	Final Density [%]
Primary trials	160	700	0.12	0.03	38.1	38
Intermediate trials	180	300	0.11	0.03	109.1	80
Advanced trials	244	300	0.10	0.03	162.7	95

**Table 4:** Pure elemental alloying powders—particle size and distribution.

	W	Ta	Mo	Nb	V
Average particle size (μm)	40	45	35	50	45
Particle size distribution (μm)	20–55	25–45	20–40	28–70	20–50

**Table 5:** Average chemical compositions of samples produced by LPBF and AMF processes.

	W	Ta	Mo	Nb	V
LPBF sample	20.1 ± 3.0	23.9 ± 5.2	18.6 ± 1.3	18.4 ± 1.1	18.9 ± 1.9
AMF sample	18.8 ± 2.9	23.1 ± 1.2	19.7 ± 1.5	17.6 ± 2.1	20.8 ± 3.5

**Table 6:** Local chemical compositions (at. %) by EDS analysis, related to Figure 4a.

	W	Ta	Mo	Nb	V
**Area 1**	37.6 ± 0.4	11.9 ± 2.4	11.5 ± 0.5	20.6 ± 0.5	18.4 ± 0.2
**Area 2**	15.0 ± 1.0	24.8 ± 5.3	19.3 ± 0.6	19.4 ± 0.4	21.6 ± 0.2

**Table 7:** Local chemical compositions (at. %) by EDS analysis; related to Figure 5c.

	W	Ta	Mo	Nb	V
**Point 1**	22.7 ± 2.1	22.0 ± 3.6	25.0 ± 0.4	16.7 ± 0.3	13.6 ± 0.3
**Point 2**	0 ± 0	51.9 ± 4.1	0 ± 0	7.4 ± 0.2	40.8 ± 0.2
**Point 3**	0 ± 0	4.2 ± 1.2	0.7 ± 0.3	28.1 ± 0.4	66.9 ± 0.5

**Table 8:** Local chemical compositions (at. %) by EDS analysis, related to Figure 6a.

	W	Ta	Mo	Nb	V
**Point 1**	7.9	27.1	10.1	21.7	33.2
**Point 2**	26.6	22.4	21.1	18.4	11.7

**Table 9:** Mean densities and hardness of HEa WTaMoNbV obtained by LPBF vs. its counterpart alloy produced by AMF process.

	Mean Densities (gr/cm^3^)	Hardness (HV10)
LPBF sample	10.83	674 ± 48
AMF sample	11.01	719 ± 53

**Table 10:** Statistical deviations of the mechanical properties of the printed WTaMoNbV HEA and reference AMF WTaMoNbV HEA.

	HEA WTaMoNbV Produced by LPBF	HEA WTaMoNbV HEA Produced by AMF
Ultimate Compressive Strength (UCS) [MPa]	1391 ± 166	1107 ± 43
Young’s modulus [GPa]	15 ± 2	21 ± 3

**Table 11:** Electrochemical parameters derived from polarization curves shown in Figure 10 along with Tafel extrapolation for evaluating the corrosion rate.

	LPBF Sample	AMF Sample
E_Corr_ (v)	−0.126	−0.085
I_Corr_ (μA)	1.759	2.936
I passivation (mA)	0.020	0.012
Corrosion rate (mmpy)	0.0014	0.0014

## Data Availability

Experimental data from this study are available from the corresponding author upon reasonable request.
